# Supplementary material for: A Likelihood Approach for Real-Time Calibration of Stochastic Compartmental Epidemic Models
Source: PLoS Comput Biol. 2017 Jan 17;13(1):e1005257. doi: 10.1371/journal.pcbi.1005257 (PMC5240920; doi:10.1371/journal.pcbi.1005257)
Supplement: S1 File — (TAR.GZ) [file pcbi.1005257.s014.tar.gz › HSPH_Online-SI-Revision/IPoi09/n100-severe/n100-severe_BENCH_table8.pdf]

1	R0	{1.65278, 9.06793}	{2.7811, 19.6712}	{{2.7811, 19.6712}, {2.7811, 19.6712}}	{68.268, 116.932}	inf	1287.	7489.43	{4089., 11 423.}	481.93	0.706184	newly	875.	2386.57	{1296., 3732.}	172.75	0.372557	Reff	0.583613	0.295107	{0.242901, 0.327419}	51.7074	ar	66 342.	92 343.4	{92 163., 92 489.}	39.1929
2	R0	{1.4321, 3.80644}	{2.50507, 9.73218}	{{2.50507, 9.73218}, {2.50507, 9.73218}}	{74.9228, 155.677}	inf	47.	696.145	{271., 1008.}	1381.16	1.05904	newly	65.	391.339	{157., 598.}	502.06	0.665813	Reff	0.65732	0.273407	{0.265713, 0.28142}	58.4058	ar	54 217.	89 344.6	{89 124., 89 559.}	64.7907
3	R0	{1.68388, 4.76028}	{2.93653, 10.872}	{{2.93653, 10.872}, {2.93653, 10.872}}	{74.3907, 128.39}	inf	45.	649.492	{333., 1268.}	1343.32	1.08821	newly	41.	323.574	{167., 653.}	689.204	0.826297	Reff	0.534935	0.19296	{0.186851, 0.200653}	63.9284	ar	68 299.	93 586.3	{93 426., 93 765.}	37.0244
4	R0	{1.66014, 6.95383}	{2.95209, 14.037}	{{2.95209, 14.037}, {2.95209, 14.037}}	{77.8217, 101.86}	inf	535.	1690.47	{898., 3272.}	215.975	0.433729	newly	463.	694.145	{368., 1348.}	61.05	0.166659	Reff	0.564215	0.198081	{0.190616, 0.209274}	64.8927	ar	66 820.	93 710.9	{93 555., 93 857.}	40.2438
5	R0	{1.69984, 11.2038}	{2.76795, 19.6041}	{{2.76795, 19.6041}, {2.76795, 19.6041}}	{62.8359, 74.9772}	inf	2209.	6244.98	{3061., 9888.}	182.706	0.385896	newly	1193.	1972.13	{927., 3181.}	74.8561	0.197902	Reff	0.580631	0.277549	{0.238265, 0.322272}	52.1988	ar	68 462.	92 211.6	{92 022., 92 408.}	34.6902
6	R0	{1.65876, 6.66217}	{2.86001, 13.5542}	{{2.86001, 13.5542}, {2.86001, 13.5542}}	{72.4186, 103.45}	inf	667.	1748.84	{766., 2684.}	162.361	0.339784	newly	562.	739.279	{324., 1139.}	58.2055	0.182352	Reff	0.560711	0.213608	{0.20329, 0.222423}	61.904	ar	67 105.	92 998.7	{92 780., 93 186.}	38.5868
7	R0	{1.69966, 5.89996}	{2.79531, 12.1249}	{{2.79531, 12.1249}, {2.79531, 12.1249}}	{64.4629, 105.508}	inf	268.	1701.87	{881., 2454.}	535.025	0.73914	newly	239.	792.583	{416., 1161.}	231.625	0.456422	Reff	0.52426	0.224312	{0.214875, 0.23243}	57.2136	ar	69 396.	92 457.4	{92 317., 92 603.}	33.2315
8	R0	{1.53428, 6.3734}	{2.8833, 17.1847}	{{2.8833, 17.1847}, {2.8833, 17.1847}}	{87.9253, 169.632}	inf	613.	5252.26	{2516., 9575.}	756.813	0.86516	newly	560.	1849.28	{886., 3385.}	230.228	0.447961	Reff	0.635852	0.241984	{0.215469, 0.277979}	61.9434	ar	59 855.	93 197.4	{93 009., 93 386.}	55.7052
9	R0	{1.58939, 3.48608}	{2.44736, 9.16315}	{{2.44736, 9.16315}, {2.44736, 9.16315}}	{53.9811, 162.85}	inf	3.	837.95	{291., 1605.}	27 831.7	2.32938	newly	4.	493.017	{164., 942.}	12 225.4	1.97233	Reff	0.594845	0.288206	{0.280076, 0.295763}	51.5494	ar	62 575.	88 567.3	{88 364., 88 746.}	41.5378
10	R0	{1.58528, 5.5593}	{2.73737, 17.9172}	{{2.73737, 17.9172}, {2.73737, 17.9172}}	{72.6742, 222.292}	inf	262.	6223.6	{2857., 10 921.}	2275.42	1.30464	newly	240.	2133.58	{971., 3735.}	788.992	0.874575	Reff	0.591785	0.285291	{0.242887, 0.337381}	51.9777	ar	63 039.	91 915.1	{91 759., 92 090.}	45.8067
11	R0	{1.58305, 4.62721}	{2.9884, 12.8326}	{{2.9884, 12.8326}, {2.9884, 12.8326}}	{88.7748, 177.329}	inf	119.	1572.	{818., 2500.}	1221.01	1.05815	newly	130.	690.968	{350., 1087.}	431.514	0.662122	Reff	0.585079	0.190691	{0.184414, 0.197533}	67.4077	ar	63 222.	93 983.5	{93 851., 94 123.}	48.6563
12	R0	{1.70234, 9.34626}	{2.78018, 19.6665}	{{2.7811, 19.6712}, {2.7811, 19.6712}}	{63.3152, 110.421}	inf	1435.	7322.56	{3690., 11 423.}	410.283	0.647405	newly	887.	2335.76	{1122., 3732.}	163.333	0.355504	Reff	0.572735	0.293337	{0.241455, 0.327419}	51.269	ar	68 213.	92 339.6	{92 163., 92 505.}	35.3695
13	R0	{1.68556, 9.74954}	{2.76795, 19.6041}	{{2.76795, 19.6041}, {2.76795, 19.6041}}	{64.2155, 101.077}	inf	1573.	6244.98	{3061., 9888.}	297.011	0.533363	newly	954.	1972.13	{927., 3181.}	109.595	0.260416	Reff	0.561814	0.277549	{0.238265, 0.322272}	50.5978	ar	68 426.	92 211.6	{92 022., 92 408.}	34.761
14	R0	{1.59876, 9.78897}	{2.79269, 19.984}	{{2.79269, 19.984}, {2.79269, 19.984}}	{74.6785, 104.148}	inf	2224.	6576.88	{3282., 10 483.}	195.723	0.403491	newly	1422.	2077.23	{1025., 3278.}	61.7628	0.168185	Reff	0.640335	0.298221	{0.236485, 0.3163}	59.1673	ar	63 553.	92 427.9	{92 255., 92 594.}	45.4343
15	R0	{1.43577, 5.12819}	{2.51743, 15.191}	{{2.51743, 15.191}, {2.51743, 15.191}}	{75.3366, 196.225}	inf	365.	3543.9	{1465., 5711.}	870.932	0.901708	newly	410.	1410.34	{580., 2245.}	243.985	0.448812	Reff	0.682565	0.303424	{0.27654, 0.321778}	55.5465	ar	53 259.	89 452.	{89 239., 89 637.}	67.9567
16	R0	{1.58866, 8.73191}	{2.77663, 19.6484}	{{2.76795, 19.6041}, {2.7811, 19.6712}}	{74.7781, 125.018}	inf	1770.	7429.4	{3510., 11 668.}	319.74	0.562539	newly	1205.	2363.46	{1122., 3724.}	99.8288	0.245885	Reff	0.609442	0.295201	{0.241455, 0.339434}	53.3659	ar	64 349.	92 290.8	{92 095., 92 456.}	43.4223
17	R0	{1.43012, 3.8163}	{2.47628, 10.2848}	{{2.47628, 10.2848}, {2.47628, 10.2848}}	{73.1519, 169.497}	inf	129.	1365.1	{519., 2038.}	958.215	0.903657	newly	198.	740.484	{271., 1084.}	273.982	0.446269	Reff	0.671055	0.287044	{0.275659, 0.298045}	57.225	ar	53 353.	88 960.6	{88 714., 89 142.}	66.7396
18	R0	{1.47965, 5.16472}	{2.45839, 11.6881}	{{2.45839, 11.6881}, {2.45839, 11.6881}}	{66.1467, 126.307}	inf	307.	2116.28	{748., 4634.}	589.342	0.702035	newly	352.	1042.66	{364., 2321.}	198.184	0.341236	Reff	0.658962	0.300334	{0.280797, 0.332276}	54.4231	ar	56 086.	88 717.5	{88 477., 88 929.}	58.1812
19	R0	{1.57191, 5.2314}	{2.86001, 13.5542}	{{2.86001, 13.5542}, {2.86001, 13.5542}}	{81.9449, 159.093}	inf	180.	1748.84	{766., 2684.}	871.576	0.907892	newly	174.	739.279	{324., 1139.}	324.873	0.546734	Reff	0.584248	0.213608	{0.20329, 0.222423}	63.4388	ar	63 119.	92 998.7	{92 780., 93 186.}	47.3386
20	R0	{1.54435, 5.32911}	{2.93371, 17.1903}	{{2.93371, 17.1903}, {2.93371, 17.1903}}	{89.9641, 222.574}	inf	264.	4720.13	{2347., 7210.}	1687.93	1.1887	newly	266.	1656.75	{812., 2574.}	522.84	0.728354	Reff	0.594389	0.227721	{0.203717, 0.247693}	61.6883	ar	61 870.	93 548.3	{93 413., 93 699.}	51.2013
21	R0	{1.57932, 4.90031}	{2.79531, 12.1249}	{{2.79531, 12.1249}, {2.79531, 12.1249}}	{76.9945, 147.431}	inf	136.	1087.88	{557., 1563.}	699.914	0.83733	newly	157.	504.05	{271., 756.}	221.051	0.43976	Reff	0.576847	0.21932	{0.21194, 0.225973}	61.9796	ar	63 595.	92 457.4	{92 317., 92 603.}	45.3846
22	R0	{1.47044, 4.07694}	{2.4534, 12.3139}	{{2.4534, 12.3139}, {2.4534, 12.3139}}	{66.848, 202.038}	inf	134.	2283.1	{949., 3158.}	1603.8	1.11506	newly	178.	1080.56	{436., 1489.}	507.055	0.662718	Reff	0.652831	0.304572	{0.286484, 0.31693}	53.3461	ar	55 895.	88 634.4	{88 418., 88 871.}	58.5731
23	R0	{1.64997, 3.34546}	{2.82466, 9.2843}	{{2.82466, 9.2843}, {2.82466, 9.2843}}	{71.1946, 177.519}	inf	4.	326.6	{160., 479.}	8065.	1.83058	newly	5.	184.433	{88., 281.}	3588.67	1.48326	Reff	0.554456	0.20895	{0.203263, 0.21346}	62.3143	ar	66 404.	92 686.6	{92 521., 92 865.}	39.5798
24	R0	{1.67405, 4.50254}	{2.98388, 9.60029}	{{2.98388, 9.60029}, {2.98388, 9.60029}}	{78.2432, 113.219}	inf	61.	478.403	{256., 700.}	684.268	0.831703	newly	72.	261.129	{139., 396.}	262.679	0.494343	Reff	0.536115	0.183832	{0.178347, 0.190819}	65.7103	ar	68 017.	93 948.	{93 795., 94 112.}	38.1243
25	R0	{1.50663, 3.1286}	{2.53701, 7.70916}	{{2.53701, 7.70916}, {2.53701, 7.70916}}	{68.3897, 146.409}	inf	23.	275.302	{120., 443.}	1096.96	0.973419	newly	31.	183.254	{80., 290.}	491.142	0.666053	Reff	0.620536	0.261663	{0.255959, 0.268263}	57.8327	ar	58 823.	89 782.	{89 559., 90 004.}	52.6307
26	R0	{1.69822, 6.34992}	{2.76203, 15.0558}	{{2.76203, 15.0558}, {2.76203, 15.0558}}	{62.6426, 137.102}	inf	344.	3819.38	{1663., 6967.}	1010.29	0.967745	newly	289.	1499.45	{640., 2729.}	418.841	0.635301	Reff	0.518704	0.25067	{0.228807, 0.284351}	51.6738	ar	69 816.	92 152.	{91 982., 92 321.}	31.9927
27	R0	{1.55869, 4.92988}	{2.81617, 13.9425}	{{2.81617, 13.9425}, {2.81617, 13.9425}}	{80.6754, 182.816}	inf	156.	2395.59	{1197., 3557.}	1435.63	1.11577	newly	170.	994.951	{486., 1502.}	485.265	0.694347	Reff	0.591429	0.226792	{0.214057, 0.236755}	61.6536	ar	62 344.	92 631.6	{92 473., 92 787.}	48.5814
28	R0	{1.46085, 6.06899}	{2.63407, 13.6136}	{{2.63407, 13.6136}, {2.63407, 13.6136}}	{80.3108, 124.314}	inf	471.	2795.68	{1186., 6255.}	493.563	0.676461	newly	430.	1197.38	{494., 2685.}	178.594	0.345996	Reff	0.667083	0.267027	{0.250316, 0.299099}	59.9709	ar	55 484.	90 871.9	{90 689., 91 020.}	63.7804
29	R0	{1.63017, 6.25164}	{2.81617, 13.9425}	{{2.81617, 13.9425}, {2.81617, 13.9425}}	{72.7531, 123.021}	inf	417.	2395.59	{1197., 3557.}	474.482	0.688757	newly	364.	994.951	{486., 1502.}	173.446	0.364171	Reff	0.557469	0.226792	{0.214057, 0.236755}	59.3176	ar	66 231.	92 631.6	{92 473., 92 787.}	39.8614
30	R0	{1.45173, 5.68165}	{2.53741, 16.1789}	{{2.53741, 16.1789}, {2.53741, 16.1789}}	{74.7853, 184.757}	inf	490.	5086.46	{2599., 8165.}	938.053	0.950807	newly	473.	1912.03	{983., 3131.}	304.235	0.535355	Reff	0.663223	0.319783	{0.282135, 0.349579}	52.5324	ar	55 280.	89 740.	{89 517., 89 990.}	62.3373
31	R0	{1.55808, 2.77316}	{2.78624, 7.73801}	{{2.78624, 7.73801}, {2.78624, 7.73801}}	{78.8252, 179.032}	inf	8.	83.2881	{36., 137.}	941.102	0.940567	newly	17.	54.2712	{23., 84.}	219.84	0.426544	Reff	0.608757	0.213049	{0.20777, 0.219277}	65.0027	ar	60 940.	92 376.6	{92 163., 92 571.}	51.5862
32	R0	{1.56797, 3.90327}	{2.70795, 10.355}	{{2.70795, 10.355}, {2.70795, 10.355}}	{72.7042, 165.29}	inf	58.	804.226	{402., 1159.}	1286.6	1.08049	newly	67.	427.629	{209., 623.}	538.252	0.741663	Reff	0.594323	0.23326	{0.227576, 0.23987}	60.752	ar	62 228.	91 627.4	{91 451., 91 807.}	47.2447
33	R0	{1.70027, 5.43261}	{2.93653, 10.872}	{{2.93653, 10.872}, {2.93653, 10.872}}	{72.7096, 100.125}	inf	114.	649.492	{333., 1268.}	469.73	0.684513	newly	106.	323.574	{167., 653.}	205.258	0.413775	Reff	0.533137	0.19296	{0.186851, 0.200653}	63.8067	ar	68 798.	93 586.3	{93 426., 93 765.}	36.0306
34	R0	{1.61668, 6.86192}	{2.7811, 19.6712}	{{2.7811, 19.6712}, {2.7811, 19.6712}}	{72.0254, 186.672}	inf	584.	7489.43	{4089., 11 423.}	1182.44	1.04935	newly	504.	2386.57	{1296., 3732.}	373.525	0.612135	Reff	0.585885	0.295107	{0.242901, 0.327419}	51.8688	ar	64 693.	92 343.4	{92 163., 92 489.}	42.7409
35	R0	{1.55955, 3.45071}	{2.68628, 9.02342}	{{2.68628, 9.02342}, {2.68628, 9.02342}}	{72.2471, 161.495}	inf	18.	370.145	{177., 583.}	1956.36	1.24626	newly	21.	218.323	{99., 332.}	939.631	0.947761	Reff	0.596107	0.233014	{0.228495, 0.238031}	60.9107	ar	61 783.	91 437.7	{91 257., 91 604.}	47.9981
36	R0	{1.65068, 4.91307}	{2.82285, 12.7912}	{{2.82285, 12.7912}, {2.82285, 12.7912}}	{71.0113, 160.35}	inf	177.	2728.1	{1221., 4490.}	1441.3	1.101	newly	195.	1218.9	{523., 2013.}	525.078	0.707944	Reff	0.557286	0.22866	{0.212307, 0.242483}	58.969	ar	66 453.	92 696.5	{92 522., 92 863.}	39.4919
37	R0	{1.46131, 3.06714}	{2.54659, 8.24572}	{{2.54659, 8.24572}, {2.54659, 8.24572}}	{74.2676, 168.841}	inf	14.	280.222	{121., 518.}	1901.59	1.20865	newly	26.	176.063	{81., 330.}	577.167	0.736249	Reff	0.636678	0.261086	{0.255168, 0.266552}	58.9925	ar	56 452.	89 847.	{89 663., 90 069.}	59.1564
38	R0	{1.71034, 5.2546}	{2.9884, 12.8326}	{{2.9884, 12.8326}, {2.9884, 12.8326}}	{74.7255, 144.216}	inf	137.	1572.	{818., 2500.}	1047.45	0.99698	newly	136.	690.968	{350., 1087.}	408.065	0.642527	Reff	0.519003	0.190691	{0.184414, 0.197533}	63.2582	ar	69 816.	93 983.5	{93 851., 94 123.}	34.616
39	R0	{1.43565, 5.57989}	{2.44597, 12.182}	{{2.44101, 12.0941}, {2.4534, 12.3139}}	{70.3734, 118.32}	inf	344.	1303.66	{448., 2445.}	279.265	0.473833	newly	352.	617.328	{217., 1149.}	94.1867	0.239455	Reff	0.663615	0.294167	{0.282705, 0.305712}	55.672	ar	54 407.	88 511.6	{88 279., 88 805.}	62.6842
40	R0	{1.40327, 4.00764}	{2.28593, 9.04943}	{{2.28593, 9.04943}, {2.28593, 9.04943}}	{62.9002, 125.804}	inf	152.	1159.82	{310., 2166.}	663.038	0.709474	newly	210.	711.109	{180., 1278.}	243.835	0.377082	Reff	0.680319	0.333552	{0.319047, 0.342958}	50.9712	ar	51 976.	86 019.3	{85 751., 86 262.}	65.4982
41	R0	{1.63612, 4.26741}	{2.82466, 9.2843}	{{2.82466, 9.2843}, {2.82466, 9.2843}}	{72.6438, 117.563}	inf	56.	326.6	{160., 479.}	483.214	0.684452	newly	65.	184.433	{88., 281.}	184.564	0.373232	Reff	0.573476	0.20895	{0.203263, 0.21346}	63.5642	ar	65 029.	92 686.6	{92 521., 92 865.}	42.5311
42	R0	{1.57426, 6.50271}	{2.95893, 18.8076}	{{2.96978, 18.8857}, {2.96978, 18.8857}}	{87.9568, 189.226}	inf	569.	6689.88	{3050., 15 226.}	1075.73	0.99505	newly	480.	2173.78	{1006., 4966.}	352.871	0.577222	Reff	0.602202	0.244789	{0.204974, 0.324211}	59.619	ar	62 522.	93 796.1	{93 636., 93 994.}	50.021
43	R0	{1.37818, 5.37636}	{2.29055, 13.7413}	{{2.29055, 13.7413}, {2.29055, 13.7413}}	{66.2011, 155.587}	inf	520.	3746.8	{1427., 7108.}	620.538	0.763999	newly	563.	1651.95	{628., 3121.}	195.117	0.374523	Reff	0.701742	0.37127	{0.332176, 0.411887}	48.5247	ar	50 098.	86 107.	{85 809., 86 409.}	71.8771
44	R0	{1.66606, 4.92641}	{2.93597, 11.6547}	{{2.93597, 11.6547}, {2.93597, 11.6547}}	{76.2223, 136.576}	inf	140.	1227.9	{640., 2384.}	777.073	0.885978	newly	138.	586.148	{297., 1128.}	324.745	0.570175	Reff	0.551433	0.197088	{0.190838, 0.204197}	64.2589	ar	67 066.	93 584.1	{93 441., 93 733.}	39.5403
45	R0	{1.623, 5.9449}	{2.86001, 13.5542}	{{2.86001, 13.5542}, {2.86001, 13.5542}}	{76.2175, 127.997}	inf	438.	2608.87	{1157., 3990.}	495.632	0.697496	newly	424.	1117.46	{495., 1703.}	163.977	0.343569	Reff	0.585189	0.22097	{0.206464, 0.233892}	62.2395	ar	64 554.	92 998.7	{92 780., 93 186.}	44.0634
46	R0	{1.65235, 5.51234}	{2.96947, 15.963}	{{2.96947, 15.963}, {2.96947, 15.963}}	{79.7119, 189.587}	inf	274.	4617.48	{2157., 8352.}	1585.21	1.15447	newly	254.	1722.65	{784., 3117.}	578.207	0.755944	Reff	0.558213	0.22155	{0.19744, 0.259324}	60.3109	ar	66 509.	93 829.	{93 704., 93 974.}	41.0771
47	R0	{1.62138, 8.82208}	{2.97157, 19.3547}	{{2.97157, 19.3547}, {2.97157, 19.3547}}	{83.2741, 119.389}	inf	1228.	5871.52	{2690., 10 052.}	378.136	0.606812	newly	834.	1859.42	{841., 3195.}	124.604	0.280766	Reff	0.585561	0.23369	{0.203612, 0.270948}	60.0912	ar	65 598.	93 849.1	{93 703., 93 993.}	43.067
48	R0	{1.64073, 6.69261}	{2.86001, 13.5542}	{{2.86001, 13.5542}, {2.86001, 13.5542}}	{74.3133, 102.525}	inf	581.	2608.87	{1157., 3990.}	349.031	0.574794	newly	486.	1117.46	{495., 1703.}	131.974	0.292006	Reff	0.561064	0.22097	{0.206464, 0.233892}	60.6158	ar	66 438.	92 998.7	{92 780., 93 186.}	39.9781
49	R0	{1.65556, 3.796}	{2.9751, 8.95585}	{{2.9751, 8.95585}, {2.9751, 8.95585}}	{79.7035, 135.929}	inf	19.	240.339	{121., 338.}	1164.94	1.03862	newly	19.	137.629	{68., 204.}	624.363	0.793977	Reff	0.553586	0.183772	{0.180321, 0.187104}	66.8034	ar	66 575.	93 878.8	{93 754., 94 012.}	41.0121
50	R0	{1.56828, 3.65713}	{2.82466, 9.2843}	{{2.82466, 9.2843}, {2.82466, 9.2843}}	{80.112, 153.868}	inf	15.	326.6	{160., 479.}	2077.33	1.25655	newly	19.	184.433	{88., 281.}	870.702	0.903477	Reff	0.584592	0.20895	{0.203263, 0.21346}	64.257	ar	62 761.	92 686.6	{92 521., 92 865.}	47.6818
